# Multi-Stage Audit to Improve Accuracy of MDT Meeting Documentation on a General Adult Ward

**DOI:** 10.1192/bjo.2025.10654

**Published:** 2025-06-20

**Authors:** Charles Pope, Raghu Vutla, Pankajam Nagarajan, Sophia Kim, Isabella Tea

**Affiliations:** 1 Leeds and York Partnership Foundation Trust, Leeds, United Kingdom; 2 South West Yorkshire Foundation Trust, Wakefield, United Kingdom

## Abstract

**Aims:** Stanley Ward is a 30-bed acute male ward in Wakefield. Quality of MDT meeting documentation was poor. We audited documentation quality in three cycles from March to November 2024, with interventions.

**Methods:** We set standards from the RCPsych CCQI Standards for Inpatient Mental Health Services and four domains of interest – capacity to consent to treatment, physical health, medications and leave. For each MDT meeting we noted whether each domain was documented fully, partially, or blank.

We audited for one-week periods in early and late March 2024, and for two-week periods in July and November 2024.

As first intervention, between early March and late March, we created an inpatient list for use by the ward doctors, including information for each domain, which could be easily copied in and updated. We continued to use the list after the audit.

As second intervention, between July and November 2024, we created a training video for new doctors, which described a psychiatric MDT and how to document. The video was interactive – a filmed simulated MDT, with audience invited to document as though present. This was shown at departmental teaching.

**Results:** Early March confirmed poor quality:

Capacity – 35% full, 30% partial, 35% blank.

Physical – 0% full, 46% partial, 54% blank.

Medications – 22% full, 16% partial, 62% blank.

Leave – 20% full, 11% partial, 69% blank.

Following first intervention, re-audit in late March showed improvement:

Capacity – 59% full, 2% partial, 39% blank.

Physical – 41% full, 32% partial, 25% blank.

Medications – 82% full, 0% partial, 18% blank.

Leave – 68% full, 2% partial, 29% blank.

Re-audit in July showed mixed **Results:**

Capacity – 20% full, 49% partial, 31% blank.

Physical – 48% full, 40% partial, 12% blank.

Medications – 91% full, 8% partial, 1% blank.

Leave – 71% full, 11% partial, 18% blank.

Following second intervention, results remained mixed, better than the start:

Capacity – 45% full, 35% partial, 42% blank.

Physical – 42% full, 16% partial, 42% blank.

Medications – 68% full, 19% partial, 13% blank.

Leave – 77% full, 0% partial, 23% blank.

**Conclusion:** Ensuring consistent improvement in quality for MDT documentation is a challenge, complicated by limited meeting time, and rotations of trainees new to psychiatry.

We have tried different interventions, including strategies to improve access to information, and producing training material.

Our next intervention is to create a training pack for new doctors in the department, which includes the interactive video. We will then re-audit.

